# Macro-level socioeconomic factors and mental health in midlife and older adults in China: a multilevel analysis

**DOI:** 10.3389/fpubh.2024.1435263

**Published:** 2024-12-23

**Authors:** Guannan Li, Gindo Tampubolon, Asri Maharani, Chenglin Tu

**Affiliations:** ^1^Global Development Institute, School of Environment, Education and Development, The University of Manchester, Manchester, United Kingdom; ^2^Division of Nursing, Midwifery and Social Work, School of Health Sciences, Faculty of Biology, Medicine and Health, The University of Manchester, Manchester, United Kingdom; ^3^Guangzhou Development Academy, Guangzhou University, Guangzhou, China

**Keywords:** mental health, depression, cognitive function, life satisfaction, economic development, economic inequality, health infrastructure

## Abstract

**Objective:**

This study explores the associations between four macro-level factors—Economic Development (ED), Economic Inequality (EI), Governmental Willingness and capacities to invest in Public Health (GWPH) and Public Health-Related Infrastructures (PHRI)—and three mental health indicators: depressive symptoms, cognitive function and life satisfaction, among middle-aged and older adults in China.

**Materials and methods:**

We obtained individual-level data from the Harmonised China Health and Retirement Longitudinal Survey (H-CHARLS) 2018 and acquired our provincial-level data from the Chinese Statistical Yearbook. Two-level linear mixed models are used to examine the associations. Supplementary analyses are carried out to test the robustness of the study.

**Results:**

There are provincial variations in macro-level factors. Depressive symptoms and cognitive functions also vary across provinces, whereas life satisfaction does not. We find that ED contributes to better depressive status. EI contributes to worse cognitive functions and life dissatisfaction. GWPH and PHRI are not associated with mental health.

**Conclusion:**

The study suggests that macro-level ED contributes to better depressive status. EI and potential systematic inequality lead to worsened cognitive functions and life dissatisfaction. It is too soon to generalise whether institutional factors like GWPH and PHRI are good or bad for mental health, but the current public health system in China does not adequately support mental and cognitive health.

## 1 Introduction

Three overarching ideas have been proposed to understand health disparities at a macro-socioeconomic level (1). Economic development (ED) contributes to better health outcomes, while economic inequality (EI) detracts from these benefits, although institutional factors, such as investment in health infrastructure, can mitigate these effects (2–4).

Controversy persists over whether ED positively or negatively affects health outcomes. In general, ED enables governments to raise resources for public health by investing in health infrastructures, which make basic health services possible. Such services have been crucial to eliminating communicable diseases and improving maternal and child mortality (5). Health investment also buffers the negative effects arising from EI (2). Nevertheless, ED could lead to increased chronic diseases due to urbanisation, which shifts lifestyles towards unhealthy diets and sedentary behaviours, particularly in developing countries (6, 7).

Although ED, in theory, gives authorities opportunities to raise funds to invest in public health, governments with different socio-economic ideologies do not always have the incentive to undertake such actions. For instance, under the notion of decentralisation, the central government tends to devolve responsibility for health services to the local, including implementation of local plans, procurement of equipment, financing and evaluation (8–11). Fiscal decentralisation is usually measured by the ratio of local public expenditure on health to total local public expenditure. Aligned with the total government expenditure on health, this measurement reflects the willingness and capacities of local governments to distribute their funds to the health sector (12). Hence, it could contribute to better health.

The impact of EI on health is a topic of debate as well. On the one hand, some research suggests that EI offsets the benefits of economic progress, leading to poorer health outcomes (3). EI may harm health due to relative deprivation, impacting mental well-being and social cohesion, causing pressures and physiological changes (13). On the other hand, researchers argue that there is little evidence to connect economic inequality to health (14), especially within emerging economies. Further, studies indicate that ED is more important than EI in determining health, particularly across developing countries (15, 16), where there is either a lack of health services or competent governments, sometimes both.

To sum up, the literature underscores two important macro-level factors intertwined with overall health: economic development (ED) and economic inequality (EI). The effects on health are mediated by institutional factors, such as governmental willingness and capacity to invest in public health and public health-related investment. However, the specific pathways and how they influence health are still tentative.

Over decades, China has experienced a remarkable development. Such development contributes to a shift in epidemiological patterns, transitioning from maternal, child, and infectious diseases to chronic non-communicable illnesses, including mental and cognitive health disorders. It has been documented that the prevalence of mental health disorders in China keeps increasing, particularly for the middle-aged and older adults (5). This age cohort is vulnerable because middle age marks a stage of life where limited physical functioning and chronic diseases start to become prevalent. Moreover, this period is associated with increasing healthcare needs. Individuals who fall in this age category are vulnerable to various socio-economic disadvantages, particularly those stemming from macro-level factors (1, 17). Despite this growing concern, research exploring the relationship between macro-level factors and health remains limited, with studies specifically addressing mental health being even scarcer.

Among the few, Wang and Granados (18) examine the relationship between economic growth and three mental health indicators, respectively. The cross-sectional study finds that higher rates of economic growth are associated with poorer depressive status, worse cognitive capacities and life dissatisfaction. These findings stand in contrast with the evidence found in Western Europe, North America and Japan, where it is slower economic growth associated with worse mental health (18). They propose that the rapid economic growth tends to heighten insecurity, as different skill sets are differently rewarded, and inequality creates winners and losers in the establishment of a market-oriented economy (18–20). In addition, pollution and congestion, driven by fast industrialisation and urbanisation, also have detrimental effects on depressive status, but these effects are either small or not casual (21). The study also highlights that mental health is worse at lower income levels. However, the primary focus of the study is on examining mental health in its entirety, without delving into the details of how ED separately influences cognitive abilities and life satisfaction. Further, the study did not adjust the effect of EI on mental health.

Different from depression, which is thought to develop through a “psycho-neuro-endocrine” pathway (22), cognitive functions decline throughout adulthood. Such decline begins in individuals’ mid-twenties and becomes more prominent during later midlife, especially after the age of 50 (23–26). Although cognitive function tends to decline with chronological age, recent studies find that younger cohorts reported a higher level of cognitive function compared to their previous generation. Such a phenomenon is called the Flynn effect (25). It has been suggested that ED improves overall quality of life, such as access to a hygienic living environment and nutritious foods, which contribute to better cognitive functions. ED also made basic social services possible, including basic health services and compulsory schooling. Higher educational attainment has been repeatedly found contribute to early cognitive stimulation of the brain, which in turn may influence later cognitive function (27), despite a roughly 16-year lagged effect (28). However, obesity, the prevalence of which rises with ED, is negatively related to cognitive performance (29). In contrast, higher degrees of metropolitan-level income inequality, measured by the Gini coefficient, predict lower level of cognitive functions with a lag period of 16–18 years, regardless of gender, race/ethnicity, or net wealth (28). Such a pathway coincides with the pattern of epidemiological transition that China has experienced in recent decades. We thus posit that macro-level factors, such as ED and IE, could affect the cognitive function of middle-aged and older adults in China.

Life satisfaction or happiness, these subjective well-being measures reflect a person’s cognitive and affective evaluation of one’s own life (30). Positive emotions are associated with higher levels of cognitive mechanics in later life (31). It is statistically meaningful to examine life satisfaction as an independent indicator of mental health (32).

Previous studies find income inequality, as an indicator of persistent unfairness, has a negative effect on life satisfaction (33). Such negative effects have been replicated based on Germany (34), European (35), American (36) and Japanese data (37). In contrast, empirical evidence also indicates a positive association between income inequality and happiness in Canada (38), the United Kingdom (39) and Japan (40): people may appreciate inequality if it signals social mobility, a phenomenon also called the ‘tunnel effect’ (41). Yet another Russian study found no relationship between happiness and income inequality (42).

Similarly, research on income inequality and happiness in China yields contrary findings. Smyth and Qian's (43) study shows that perceived income inequality lowers happiness in urban China, with varying impacts based on income levels. Knight and Gunatilaka (44) find that a higher Gini coefficient in rural counties increases happiness due to the “demonstration effect.” Jiang, Lu and Sato (45) find a positive correlation between city-level Gini coefficients and happiness in China. In contrast, many studies suggest socio-economic development is more important than inequality in determining self-reported health status (1). For clarity, we summarise these empirical findings in the Supplementary Table S1.

In summary, there are gaps in the literature. Firstly, existing research has overlooked the examination of the relationship between macro-level factors and mental health. Among the few studies, they typically explore only the relationship between a single macro-level indicator and a single aspect of mental health, such as the relationship between inequality and cognitive impairment (28), or economic development and depression (46). Comprehensive studies that account for the effects of EI, ED and institutional factors on mental health are exceedingly rare. Second, the relationship between macro-level factors and mental health outcomes exhibits strong heterogeneities between developed and developing countries. Studying China, one of the largest developing countries, will enhance our understanding in this context.

The present study fills the gap by examining the associations between macro-level factors and mental health among middle-aged and older adults in China using several macro-level variables as the covariates and using a large and nationally representative dataset with a multilevel structure.

## 2 Materials and methods

### 2.1 Data source and study sample

Given the multi-level structure of our study, we draw on our data from different sources.

We obtain our mental health variables and individual-level covariates from the Harmonised-China Health and Retirement Longitudinal Study Version D (H-CHARLS). H-CHARLS is a longitudinal dataset that has been designed to align with a series of ageing studies worldwide, including the Health and Retirement Study (HRS) and the Survey of Health, Ageing and Retirement in Europe (SHARE). H-CHARLS provides standardised measures and improved generalisability, aiming to international collaborations and comparisons. Details of H-CHARLS can be found elsewhere (47, 48).

Although the latest data (2020) is available, to avoid potential confounding and cohort effects from the pandemic, particularly on mental health, we opt to use survey data from 2018. We obtained our provincial-level (macro-level) information across 28 provinces based on year 2018 from the Chinese Statistical Yearbook 2019, due to the lag in data administration.

We included people who are 45 years or older as our study sample. This age cohort is particularly vulnerable because middle age marks a stage of life where limited physical functioning and chronic diseases start to become prevalent. Moreover, this period is associated with increasing healthcare needs. Individuals who fall in this age category are vulnerable to various socio-economic disadvantages, particularly those stemming from macro-level factors (1).

The final analytical sample comprises 17,547 individuals across 28 provinces in China. The average age of the sample is 63.15 (SD = 9.60), and there are slightly more women than men (51.33% vs. 48.67%). Sample weights that the CHARLS team has computed for individual non-responses are applied.

### 2.2 Outcome variables: mental health

Depression is the most common forms of mental disorders. It is estimated that 5% of world population suffers from some form of depressive symptoms. Also, depression is an important predictor to many other mental disorders, such as anxiety or even suicide. Cognitive functions, likewise, are core features of mental health conditions. Cognitive impairments encompass difficulties with attention, memory recall, planning, organising, reasoning and problem-solving, losing these capacities is a precursor to dementia (49). An early study also highlights that assessing of life satisfaction as a standalone measure of mental well-being statistically merits independent examination (32). Accordingly, and following the previous study (18), we selected the indices of (1) depressive symptoms, (2) cognitive function and (3) life satisfaction as the indicators of mental health.

#### 2.2.1 Depressive symptoms

H-CHARLS used the 10-item Centre for Epidemiological Studies Depression Scale (CES-D 10) to measure depression. CES-D 10 has a possible total summary score of 0–30, and higher scores indicate a higher level of depression or poorer mental health.

Strong psychometric properties, internal consistency and validity have been demonstrated by previous studies. The CES-D 10 demonstrates strong predictive accuracy relative to the 20-item version, with good internal consistency across general, older, and multiethnic populations. It also shows acceptable to good sensitivity and specificity in detecting depression in China (50–53).

#### 2.2.2 Cognitive function

H-CHARLS used the Telephone Interview for Cognitive Status (TICS) to measure cognitive function. The interview was carried out face-to-face. The tests consist of 30 items, including orientation, serial of 7 subtraction and episodic memory tests (54, 55). TICS scores range from 0 to 30, with higher scores indicating better cognitive function.

The TICS demonstrates strong reliability (inter-rater and intra-rater coefficients of 0.89–0.98) and validity, correlating well with tools like the Mini-Mental State Examination (MMSE) and Clinical Dementia Rating (CDR). It is effective for screening dementia and mild cognitive impairment, particularly when in-person assessments are impractical. In China, TICS has been culturally adapted and validated, proving sensitive to early cognitive decline and suitable for large-scale monitoring of older adults (56, 57).

#### 2.2.3 Life satisfaction

To assess life satisfaction, respondents were asked to think about life as a whole and to rank their life satisfaction on a five-point Likert scale. We assigned 4 points for “completely satisfied,” 3 for “very satisfied,” 2 for “somewhat satisfied,” 1 for “not very satisfied,” and 0 for “not at all satisfied.” The total scores range from 0 to 4, with a higher score representing greater life satisfaction. The use of this Likert scale has been proved good internal reliability and validity by a previous study on macro-level economic growth and life satisfaction in China and worldwide (18, 38, 41, 42).

### 2.3 Provincial-level variables

In light of previous studies (1, 58), this study offers a concise summary of macro-level economic, institutional and healthcare infrastructure factors influencing the mental health of middle-aged and older adults in China. We constructed six variables drawn from the Chinese Statistical Yearbook to capture the economic situation and healthcare provision of each province. Accounting for population, they are (1) gross domestic product *per capita* (GDP *per capita*, unit: 10000 Chinese yuan), (2) ratio of urbanisation, (3) ratio of local government expenditure on health care, (4) local government general budget expenditure, (5) number of hospital beds per 1,000 population and (6) number of primary care institutions per 1,000 population.

The selection of these six macro-level variables reflects their pivotal roles in shaping the socio-economic environment, which significantly influences mental health outcomes among the ageing population in China. GDP *per capita* captures the general economic prosperity, which can impact mental well-being by improving access to resources and reducing financial stress. The urbanisation ratio reflects shifts in living environments and changes in lifestyles, potentially affecting social support networks, access to mental health services and health behaviours. Government expenditure on healthcare and the general budget illustrate the prioritisation of public health and social welfare, which are crucial for developing comprehensive mental health services. Lastly, the availability of hospital beds and primary care institutions per 1,000 population indicates the healthcare infrastructure’s capacity, influencing access to both preventive and curative mental health services. Together, these variables provide a comprehensive view of the socio-economic context and its influence on mental health.

We transformed the local government’s general budget expenditure with a logarithm. We carried out a *principal component analysis* (PCA) to avoid multicollinearity and to limit the number of macro-level variables. We then interpreted the three extracted components as representing (1) an “economic development (ED)” dimension, (2) a “governmental willingness and capacities to invest in public health (GWPH)” dimension and (3) a “public health-related infrastructure (PHRI)” dimension. The discussion on the statistics of PCA and the interpretation of results will follow later.

### 2.4 The Gini coefficients

The Gini coefficient is a key measure of economic inequality, representing the distribution of income or wealth. Since official Gini coefficients for China are not available officially, we construct provincial coefficients using data from the Chinese Statistical Yearbook. Calculating the Gini coefficient is significant for this study as it allows for quantifying economic inequality, which is known to exacerbate mental health issues such as social stress, lower life satisfaction, and limited healthcare access, particularly for disadvantaged individuals. By examining this relationship, the Gini coefficient helps establish how income and wealth disparities affect mental health outcomes in the ageing Chinese population. Additionally, understanding the role of inequality can guide targeted public health and social policy interventions to mitigate its impact on vulnerable groups. In summary, the Gini coefficient provides valuable insights into the connection between economic inequality and mental health, informing future policy development.

In the Yearbook, residential income level has been divided into seven categories for urban residents since 1987 and five categories for rural residents since 2001. As the income variables are treated as discrete variable in the Yearbook, we construct the coefficients following method once used for education inequality (59):


$$\mathrm{Gini}=\left(\frac{1}{\mu }\right)\overset{n}{\underset{n=2}{\sum }}\overset{i-1}{\underset{j=1}{\sum }}p_{i}|y_{i}-y_{j}|p_{j}$$

where, Gini is the Gini coefficients, *μ* is the average income for certain population, *n* represents the number of categories (7 for urban residents and 5 for rural residents), *y_i_* is the average income of category *_i_*, *p_i_* is the ratio of the population of category *_i_* to the total population. Based on the Gini coefficient, which represents the ratio of the area of inequality in the Lorenz curve to the area of total inequality, the following formula can be derived:


$$\mathrm{Gini}=1-\frac{1}{PW}\;\overset{n}{\underset{i=1}{\sum }}\left(W_{i-1}+W_{i}\right)P_{i}$$

Where, *P* is the total population, *W* is the total income, and *W_i_* is the cumulative income up to group *_i_*. Then we can calculate the Gini coefficients for rural area and urban area, respectively. Then, following Sundrum’s method (60), we can compute Gini coefficients for each province:


$$\mathrm{Gini}=P_{u}^{2}\frac{u_{u}}{u}\;G_{u}+P_{r}^{2}\frac{u_{r}}{u}\;G_{r}+P_{c}P_{r}\frac{u_{c}-u_{r}}{u}$$

where, *G_u_* and *G_r_* represent the Gini coefficients of urban and rural residents in a given province, respectively. *P_u_* and *P_r_* represent the population proportions of urban and rural areas, respectively. *u_u_* and *u*_r_ represent the *per capita* incomes of urban and rural areas, respectively. *u* represents the national average income. Gini coefficients are calculated using MATLAB.

### 2.5 Individual-level covariates

We included demographic covariates (age and sex) and socio-economic covariates (education background, marital status, economic status and place of residence). We also accounted for covariates related to health behaviours (alcohol consumption and smoking behaviours) and functions (difficulties in activities of daily living and the presence of comorbidities). As social insurance provide a sense of security for citizens, which has been proven as a buffer for depression (46), we include the insurance status of all participants. Specification on survey instruments can be found in Supplementary Table S2.

### 2.6 Statistical analysis

We begin by presenting descriptive statistics of the sample. Then, we report mean scores for each mental health indicator by covariate subgroup. Following this, we present thermal maps to illustrate the distribution of various macro-level factors for 28 provinces in China.

An obvious model to consider for the continuous response variable *y_ij_*, mental health indicators, is a multiple linear regression model, including age, gender, provincial-level factors, and other covariates. The model for the mental health, *y_ij_*, of individual, *_i_*, of province, *_j_*, is specified as:


(1) $$y_{ij}=\beta_{0}+\beta_{1}x_{1ij}+\ldots +\beta_{p}x_{pij}+\xi_{ij}$$

where *x_1ij_* through *x_pij_* are independent variables and covariates, and *ξ_ij_* is a residual. We assume that the mental health of individuals to provinces are uncorrelated given the observed covariates, or in other words that the residuals *ξ_ij_* and *ξ_i’j_* are uncorrelated. We can therefore split the total residual or error into two error components: *ζ_j_*, which is shared between individual of the same province, and ij, which is unique for each individual:


$$\xi_{ij}=\zeta_{j}+ij$$

Substituting for *ξ_ij_* into the multiple-regression model (1), see Equation 1, we obtain a two-level linear random-intercept model with covariates:

$$y_{ij}=\beta_{0}+\beta_{1}x_{1ij}+\ldots +\beta_{p}x_{pij}+\left(\zeta_{j}+Q_{ij}\right)$$

(2) $$=\left(\beta_{0}+\zeta_{j}\right)+\beta_{1}x_{1ij}+\ldots +\beta_{p}x_{pij}+Q_{ij}$$

This model (Equation 2) can be viewed as a regression model with an added level-2 residual *ζ_j_*, or with an individual-specific intercept *β_0_ + ζ_j_*. The random intercept *ζ_j_* can be considered a latent variable that is not estimated along with the fixed parameters *β_1_* through *β_p_*. The linear random-intercept model with covariates is an example of a linear mixed (effects) model where there are both fixed and random effects. Such a random intercept model is also called as a mixed effects model.

We employ *multiple imputations (MI) by chained equations (MICE)* to handle missing data on depressive symptoms (*N* = 1,579, 8.95%), cognitive function (*N* = 1,542, 8.7%), life satisfaction (*N* = 1,439, 8.2%) and one discrete covariate, economic status quartiles (*N* = 2,987, 17.0%), using Stata’s mi programme. We impute missing continuous variables and categorical variables by applying a predictive mean matching imputation method and a multinomial logistic model, respectively (61). Five cycles of MI are used. We use Stata 15.0 (*StataCorp, College Station, TX*) for all bivariate and multivariate analysis.

### 2.7 Supplementary analysis

To assess the robustness of our study, we recalculate Gini coefficients using household expenditure data from H-CHARLS 2018, instead of income. Household expenditure is preferred because expenditures measure economic status from the perspective of quality of life, which is potentially linked to mental health. Income may not capture economic status from sources like real estate, while expenditure is less affected by sporadic income shocks (62–65). Moreover, due to substantial missing income data in H-CHARLS, expenditure provides a more reliable metric.

To accurately reflect economic status, we exclude the annual automobile expenditure, which, as is a one-off payment, is likely to overestimate household economic status in the surveyed year. Similarly, we retained fitness and care expenditures but excluded medical service expenditures to avoid overestimation. In China, people of lower socioeconomic status tend to pay more for medical services due to the healthcare reimbursement and insurance system (66).

Using Stata 15.0’s INEQDECO package, we then calculated the Gini coefficients for each of the 28 provinces and examined the relationship between macro-level factors and mental health indicators.

Last, in order to evaluate the rationality and accuracy of the imputed data, we run a multiple imputation of 10 cycles to compare to the one of five cycles. The results are collected in the Supplementary Table S3.

## 3 Results

Table 1 shows the number and percentage of observations in each covariate subgroup and summarises the mean score for each mental health indicator by covariate subgroup in the 2018 survey. In general, people who are older, female, rural residents, not married, economically disadvantageous, less educated, have no insurance of any kind, do not drink, do not smoke, have more comorbidities and have difficulties in ADL tend to report higher mean scores of CES-D 10 and lower mean scores of TICS, indicating poorer mental health. While life satisfaction mean scores show no clear differences across covariate subgroups.

**Table 1:** Descriptive statistics of the sample and mean score (standard deviation) of each mental health indicator by covariate subgroup, H-CHARLS 2018.

	N (%)	Depressive symptoms	Cognitive functions	Life satisfaction
Age				
45–49	813 (4.64)	7.36 (0.21)	15.84 (0.22)	3.20 (0.03)
50–59	6,006 (34.31)	8.27 (0.08)	14.24 (0.08)	3.22 (0.01)
60–69	6,413 (36.64)	8.60 (0.09)	10.92 (0.08)	3.26 (0.01)
70–79	3,171 (18.11)	8.95 (0.13)	9.46 (0.12)	3.29 (0.02)
80 or older	1,102 (6.3)	8.33 (0.23)	6.11 (0.21)	3.36 (0.03)
Gender				
Male	8,540 (48.67)	7.35 (0.07)	12.88 (0.07)	3.28 (0.01)
Female	9,007 (51.33)	9.55 (0.08)	10.85 (0.08)	3.23 (0.01)
Residence				
Urban	6,972 (39.73)	7.36 (0.08)	13.67 (0.08)	3.28 (0.01)
Rural	10,575 (60.27)	9.20 (0.07)	10.66 (0.07)	3.24 (0.01)
Marital status				
Married/live together	14,745 (84.03)	8.20 (0.05)	12.38 (0.06)	3.26 (0.01)
Single/divorced/separated/widows	2,802 (15.97)	10.09 (0.15)	8.68 (0.13)	3.19 (0.02)
Economic status				
Highest	3,511 (24.11)	7.93 (0.11)	13.82 (0.12)	3.29 (0.01)
Middle-high	3,607 (24.77)	8.41 (0.11)	12.72 (0.11)	3.25 (0.01)
Middle-low	3,679 (25.27)	8.68 (0.11)	11.67 (0.11)	3.25 (0.01)
Lowest	3,763 (25.84)	9.17 (0.11)	10.18 (0.11)	3.25 (0.01)
Educational background				
College degree or higher	321 (1.83)	4.94 (0.3)	18.62 (0.27)	3.35 (0.04)
High School or equivalent	1736 (9.89)	6.54 (0.13)	16.88 (0.12)	3.25 (0.02)
Middle School	3,425 (19.52)	7.54 (0.1)	15.39 (0.09)	3.25 (0.01)
Elementary school	4,739 (27.01)	8.07 (0.09)	13.72 (0.09)	3.25 (0.01)
Sishu or no school but can read	2,908 (16.57)	9.68 (0.13)	9.83 (0.11)	3.23 (0.02)
Illiterate	4,418 (25.18)	10.00 (0.11)	5.35 (0.08)	3.28 (0.01)
Insurance status				
Have insurance of any kind	16,860 (96.29)	8.45 (0.05)	11.96 (0.05)	3.26 (0.01)
Have no insurance	650 (3.71)	9.25 (0.28)	8.59 (0.28)	3.15 (0.04)
Smoking behaviours				
Do not smoke	12,735 (72.58)	8.75 (0.06)	11.67 (0.06)	3.26 (0.01)
Current smoking	4,812 (27.42)	7.77 (0.09)	12.28 (0.09)	3.25 (0.01)
Alcohol consumption				
Do not drink	11,624 (66.47)	8.96 (9.22)	11.08 (0.07)	3.24 (0.01)
At least once a month or more	5,864 (33.53)	7.16 (7.47)	13.32 (0.09)	3.28 (0.01)
Num. comorbidities				
None	3,247 (18.5)	6.25 (0.1)	12.54 (0.13)	3.38 (0.01)
1	4,052 (23.09)	7.32 (0.1)	12.08 (0.11)	3.31 (0.01)
2	3,571 (20.35)	8.30 (0.11)	11.77 (0.12)	3.27 (0.01)
3 or more	6,677 (38.05)	10.38 (0.09)	11.39 (0.09)	3.15 (0.01)
Activities of daily lives				
Have no difficulties	13,938 (80.04)	7.53 (0.05)	12.48 (0.06)	3.30 (0.01)
Have difficulties in ADL	3,476 (19.96)	12.69 (0.13)	9.00 (0.12)	3.05 (0.02)

**Figure 1:**
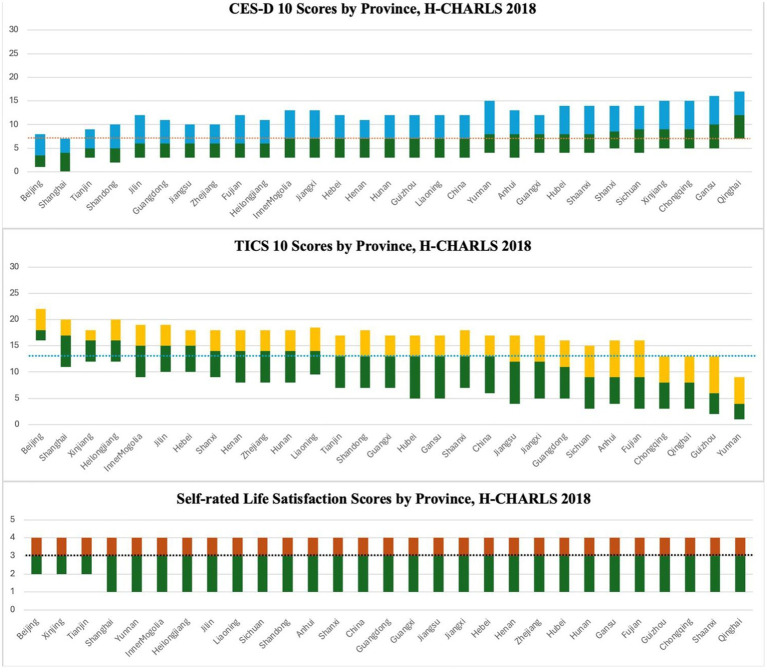
The distribution of mental health scores by province, H-CHARLS 2018. 1. For clarity, we only present the first quartile, median, and third quartile of each mental health indicator for each province, as the maximum and minimum values of CES-D 10 and TICS are 30 and 0, respectively. Those for self-rated life satisfaction are 4 and 0, respectively. 2. Dot line in each figure represents the national median mental health score.

For clarity, Figure 1 presents the distribution of each mental health score by province. Residents from Beijing and Shanghai report better mental health status. Generally, northern provinces have higher median mental health scores compared to their southern counterparts, while eastern provinces exhibit better results than those in the west. For instance, among the top 5 provinces with the lowest CES-D 10 scores (better depressive status), 4 are in the north. In contrast, 11 provinces report a higher median than the national average, with the last 5 all being southwest provinces, indicating a poorer depressive status. As for cognitive functions (TICS scores), 12 provinces report a higher median (better) than the national average, with 9 being northern provinces. All 10 provinces with lower TICS scores are located in the south. However, there are no evident disparities in life satisfaction across provinces.

The PCA produced three principal components with eigenvalues greater than one (2.58, 1.76, 0.998, respectively). The first three components account for 43.01% (PC_1_), 29.33% (PC_2_) and 14.24% (PC_3_) of the total variance, respectively (86.58% in total). PC_1_ is positively correlated to GDP *per capita* and the ratio of urbanisation (loadings are 0.58 and 0.59, respectively). As the two factors are highly associated with economic growth, we interpret the component as “Economic Development (ED).” PC_2_ is positively correlated to the total provincial government expenditure on health (log-transformed) and the ratio of total government expenditure on health to local government general budget expenditure (loadings are 0.56 and 0.58, respectively). Consistent with an early study (12), this component reflects an idea of decentralisation, a “the governmental willingness to invest in public health (GWPH).” PC_3_ is positively correlated to the number of hospital beds per 1,000 population and the number of primary care institutions per 1,000 population (loadings are 0.65 and 0.18, respectively). We interpret it as “public health-related infrastructures (PHRI).”

**Figure 2:**
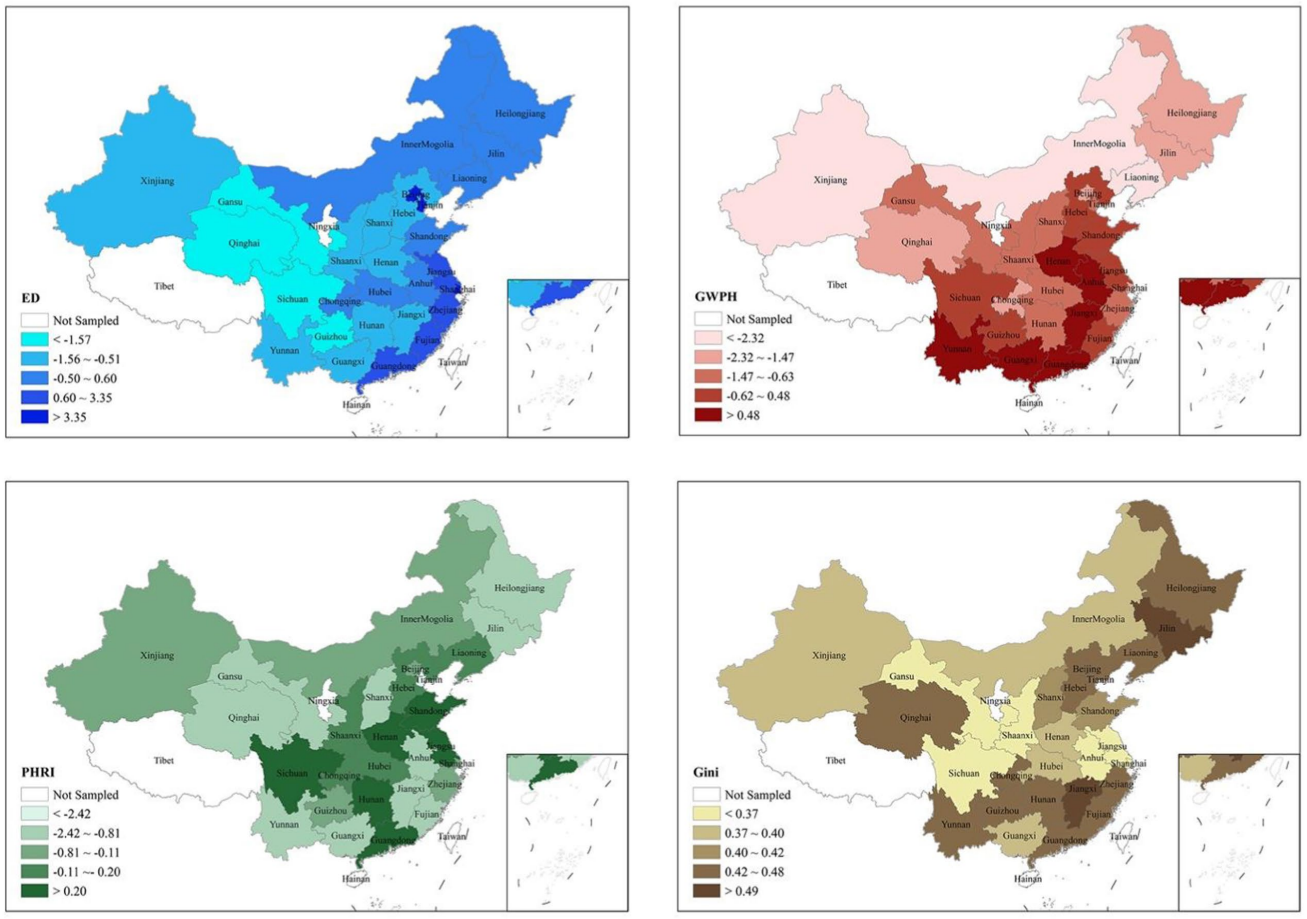
The macro-level characteristics of each province in China by 2018. ED, economic development; Gini, gini coefficients; GWPH, governmental willingness and capacities to invest in public health; PHRI, public health-related infrastructures.

Based on the PCA results, Figure 2 illustrates the macro-level characteristics of each province in China in 2018 (for actual values, see Supplementary Table S4). The coastal provinces in the southeast clearly have higher levels of ED compared to the inland provinces, with the western provinces exhibiting the lowest levels of ED in China. The three municipalities, Beijing, Tianjin and Shanghai, have the highest level of ED. Regarding EI, northeast and southern provinces report higher Gini coefficients, with Shanghai being the most equal city and in China. This result is in line with a previous study (67).

Table 2 presents regression coefficients and standard errors from two-level mixed effect models, demonstrating associations between four macro-level factors and depressive symptoms (Model 1), cognitive functions (Model 2) and life satisfaction (Model 3), respectively. We find ED is negatively associated with depressive symptoms. EI is negatively associated with cognitive function and life satisfaction. The two institutional macro-level factors, GWPH and PHRI are found not significantly associated with any mental health indicator.

**Table 2:** The coefficients and standard errors of two-level mixed effects models for mental health indicators, H-CHARLS 2018.

	Depressive symptoms	Cognitive functions	Life satisfaction
	Model 1	Model 2	Model 3
Economic development	−0.35 (0.07)***	−0.05 (0.09)	−0.01 (0.02)
Economic inequality	−0.16 (3.17)	−8.83 (4.00)*	−0.75 (0.38)*†*
GWPH	−0.08 (0.12)	−0.08 (0.15)	−0.04 (0.03)
PHRI	−0.15 (0.17)	−0.08 (0.21)	−0.01 (0.04)
Age	−0.07 (0.00)***	−0.14 (0.00)***	0.01 (0.00)***
Female (ref = male)	1.38 (0.05)***	0.32 (0.04)***	−0.03 (0.01)***
Rural resident (ref = urban)	1.14 (0.04)***	−1.08 (0.04)***	−0.02 (0.01)***
Single/divorced/separated/widows (ref = married or live together)	1.11 (0.06)***	−0.67 (0.05)***	−0.10 (0.01)***
Economic status (ref = highest)			
2^ed^ quartile (middle-high)	0.12 (0.06)***	−0.25 (0.05)***	−0.05 (0.01)***
3^rd^ quartile (middle-low)	0.29 (0.06)***	−0.5 (0.05)***	−0.07 (0.01)***
4^th^ quartile (lowest)	0.4 (0.06)***	−1.07 (0.05)***	−0.07 (0.01)***
Education background (ref = college degree or higher)			
High School	0.64 (0.16)***	−1.13 (0.13)***	−0.05 (0.02)*†*
Middle School	1.24 (0.15)***	−2.14 (0.13)***	−0.03 (0.02)
Primary School	1.49 (0.15)***	−3.46 (0.13)***	−0.01 (0.02)
Sishu/can read	2.45 (0.16)***	−6.26 (0.13)***	0.00 (0.02)
Illiterate	2.2 (0.16)***	−10.3 (0.13)***	0.05 (0.02)*†*
Have no insurance (ref = have insurance of any kind)	0.14 (0.10)*†*	−1.15 (0.09)***	−0.09 (0.01)***
Current smoker (ref = do not smoke)	0.45 (0.05)***	−0.63 (0.04)***	−0.04 (0.01)***
Drink alcohol (ref = do not drink)	−0.39 (0.05)***	0.44 (0.04)***	0.01 (0.01)*†*
Num. comorbidities (ref = none)			
1	0.78 (0.06)***	0.39 (0.05)***	−0.07 (0.01)***
2	1.54 (0.06)***	0.32 (0.05)***	−0.11 (0.01)***
3 or more	3.12 (0.06)***	0.55 (0.05)***	−0.24 (0.01)***
Have difficulties in ADL (ref = have no ADL)	3.93 (0.05)***	−0.72 (0.05)***	−0.24 ± 0.01***
Depression	n/a	−1.40 (0.04)***	n/a
Constance	5.64 (2.01)**	32.37 (2.53)***	3.38 ± 0.43***
Province-level (level 2) Variance	0.74 (0.10)	0.93 (0.13)	0.16 (0.02)
AIC/BIC	588,234/588479	559,605/559860	217,483/217728

Significant level: †10%, *5%, **1%, ***0.1%.

Instead of macro-level factors, our results suggest that individual-level covariates appear to be the most significant contributors to mental health outcomes. People who are older, female, rural residents, not married or live with anyone, economically disadvantageous, have no social insurance of any kind, currently smoking, do not consume alcohol, have more comorbidities and having difficulties in ADL, tend to report more depressive symptoms, poorer cognitive functions and life dissatisfaction, indicating worse mental health. People who are economically and educationally advantageous report fewer depressive symptoms and better cognitive functions. In contrast, individuals who are economically advantageous report higher levels of life satisfaction, but there is no significant relation between educational background and life satisfaction. In addition, although the effect is weak, consuming alcohol is positively associated with the three mental health indicators, respectively. Such reverse effect will be discussed later.

The Gini coefficients calculated based on Chinese Statistical Yearbook are correlated to those based on household expenditures from H-CHARLS (Pearson correlation coefficient, *r* = 0.08, *p* < 0.000). Our supplementary analyses demonstrate strong robustness of our models. Details see Supplementary Table S5.

## 4 Discussion

Applying nationally representative multilevel data, we explore the associations between four macro-level factors, i.e., ED, EI, GWPH and PHRI, and three mental health indicators, i.e., depressive symptoms, cognitive function and life satisfaction, respectively, among the middle-aged and older adults in China.

In line with prior research, we find there are provincial variations in ED, EI, GWPH, and PHRI (1). By using the Pearson correlation test, we find EI is weakly but positively associated with ED (pc1, *r* = 0.101, *p* < 0.001) and GWPH (pc2, *r* = 0.021, *p* < 0.05), but negatively with PHRI (pc3, *r* = −0.35, *p* < 0.000). Such results indicate that areas with higher levels of ED tend to exhibit higher levels of EI in China. Governments in less equal areas tend to have higher willingness to invest in public health. ED could play a mediating role in this context. PHRI could mitigate the negative effect of EI (2).

Likewise, depressive symptoms and cognitive functions also show variations across provinces. Beijing and Shanghai have an advantage in depressive status and cognitive functions, while northern provinces perform better than their southern counterparts. However, there is no clear difference in life satisfaction across provinces, suggesting different mental health indicators could develop along various pathways.

Our results show that *a higher level of ED is associated with better depressive status, while provincial EI is not significantly associated with depression.* Such a finding reflects a materialist pathway: ED is more important than EI in determining individual health in developing countries (1, 4). For Chinese older adults who grew up in environments with an extreme lack of medical support and clean and nutritious food, fast economic growth significantly improves the quality of life within just one generation. However, it is premature to conclude EI does not affect mental health. The reasons are twofold. First, macro-level EI may not independently predict depression, but it could mediate the known and unknown causal processes through which social class leaves its mark on individuals throughout their lives (3). Second, EI has a lagged effect on health, possibly up to 15 years. The effect of EI on depression has not yet been observed (68). Further, different mental health indicators could develop following different pathways: our results find EI contributes to worse cognitive functions.

We find *EI contributes to worse cognitive functions*, although the effect is weak. Such a weak effect may reflect a lagged effect of EI on health. One American study suggests that the effects of macro-level income inequality on individual self-rated health are strongest after a latency period of roughly 16 years, with weaker effects measured 4–12 years (69). Another study based on the U.S population finds that metropolitan-level factors better predict cognitive function than state-level factors, highlighting that the impact of macro-level factors on cognition can differ at more localised levels (28). Future research should investigate this issue or even compare the provincial-level and city-level effects on cognition with the controlled lagged effects as data becomes available in China. The effect of EI on cognitive functions could be stronger.

At the individual level, education have been consistently linked to cognitive function (25). People who are illiterate score 10 points lower on the TICS (out of 30) compared to those with a college education background (see model 2). This supports the “cognitive reserve” hypothesis, which suggests that higher educational attainment and cognitive enrichment build greater nervous system resilience against neuropathological damage (e.g., vascular damage, Alzheimer’s protein build-up, inflammation). This increased resilience raises the threshold for cognitive impairment, accounting for other macro-level socioeconomic inequalities (70, 71).

We find that *a higher level of ED does not contribute to higher levels of life satisfaction, but EI predicts life dissatisfaction* (model 3). This inconsistency between ED and happiness has been widely referred to as the Easterlin paradox (72). It states that although happiness correlates with income among and within nations at a certain point in time, it does not increase as income continues to grow. Specifically, China’s rapid economic growth has prompted an epidemiological transition. This transition over the past decades. This transition, coinciding with urbanisation and lifestyle changes, has resulted in the prevalence of chronic diseases such as cardiovascular disease, diabetes, and dementia. These chronic diseases require long-term healthcare investment, presenting fiscal challenges for most developing countries and potentially reducing life satisfaction due to socio-economic inequalities.

Two main factors associated with such socioeconomic inequalities have been reported as linked to life satisfaction in China: increasing inequality and the dissolution of the social security net during the economic reforms (73–76).

In China, the costs of health services are climbing continuously under economic restructuring and the market-oriented health service system, in turn exacerbating economic inequality (77, 78). Older adults are increasingly unable to afford health treatment or falling into poverty due to out-of-pocket payments for health services (79). In 2008, about 31% of rural older adults reported that proper medical services were not affordable (80). In urban areas, 62% of older adults reported having been ill in the past 2 weeks but did not access medical services, and 14% of them reported financial difficulties as the main reason for this (81). These findings are consistent with our model (model 3), which shows that older adults who have participated in any kind of social insurance report greater life satisfaction than those who have not.

In addition, although macro-level ED is not significantly associated with life satisfaction, individual economical advantage is associated with greater life satisfaction (model 3). As a previous study highlights that health and economic status are the most significant factors for life satisfaction among older adults (82).

We find *that PHRI and GWPH are not associated with mental health.* Throughout our models, higher levels of PHRI are found not to be associated with any mental health indicators. However, drawing the conclusion that PHRI plays no role in improving mental health may be premature. Based on the current empirical data, we reaffirm that the present provincial PHRI in China is not specifically designed to address mental health challenges and, therefore, does not improve mental health (83). Similarly, GWPH, which reflects the capacities and willingness of the local government to public health, is found to be not associated with mental health outcomes. While we acknowledge the efforts and achievements of the Chinese government in public health, the evidence suggests that the present investments are not favouring mental health.

Further, one potential reason for this lack of associations could be the uneven allocation of government health-related investment. For instance, the current medical reimbursement system favours urban residents and people working in the public sector (84, 85). Such institutional actions may widen inequality. Specifically, the New Rural Cooperative Medical Scheme (NCMS) and the Urban Resident Basic Medical Insurance (URBMI) were introduced in 2003 and 2007, respectively. Although those projects benefited the majority, we cannot ignore the fact that they also created gaps between the accessibility of medical services for rural and urban residents. Similarly, Government Medical Insurance (GMI) and Urban Employee Medical Insurance (UEMI) have created a gap between residents employed in the public and private sectors (78, 84, 85). We thus posit that economic inequality and potential systematic inequality lead to life dissatisfaction. Such topic deserves further investigations.

The present study has several limitations. First, despite self-reported life satisfaction being widely used, robust and comprehensive measurements for life satisfaction, such as the 32-item Life Satisfaction Questionnaire (LSQ-32) are preferred (86). Second, there are potential macro-level factors that have not been accounted for in our study. A direct measure of mental health support facilities at the provincial level is necessary. Air pollution could be another macro-level factor, despite its correlation with the urbanisation ratio. New theoretical frameworks are desired in the future to accommodate latent factors. Third, lagged effects on depression and cognitive health have been repeatedly reported (28, 69). The lagged associations between macro-level factors and mental health deserve further investigations. Fourth, we find that alcohol consumption is positively associated with better mental health outcomes. This result suggests the likely presence of unexamined mediators. On the one hand, alcohol consumption could be linked with economic advantages, which contribute to better health. On the other hand, the effect of alcohol consumption on mental health could be mediated by social ties and participation, which is found associated with better mental health outcomes (87). Overall, our study is constrained by data limitations.

Further, the cross-sectional design of our study has limitations. First, it can identify associations but not causality, making it unclear whether economic inequality is the cause of mental health issues. Second, the cross-sectional data captures only a snapshot in time, overlooking changes over time, particularly as individuals age. Further, it may also be influenced by confounding factors like culture or life experiences and can oversimplify complex interactions. Such cohort effects may distort results if differences between age groups or regions are not accounted for. Therefore, while informative, cross-sectional data cannot establish causal relationships or capture long-term dynamics. Longitudinal data and design are desired in the future study.

Last, we select middle-aged and older adults in China as our study sample. The interpretation and generalisation of our findings should be strictly limited to this age cohort. This caution is necessary because the same macro-level factor may influence the mental health of the younger cohort in a different way. For instance, hypothesis has been proposed that rapid economic growth increases work pressure on the younger working individuals rather than the older ones, increasing anxiety among the younger cohort (88). As for cognitive function, being a physiological capacity, it remains stable across the early stages of life, regardless of inequality. Lagged effects, referring to the delayed impact of an exposure or intervention on an outcome, may take years to manifest after inequalities have occurred. For example, the lagged effect of inequality on cognition might be more accurately assessed by considering the Gini coefficient from 15 years prior (28). Clearly, it is premature to expect individuals from the younger cohort to assess their overall life satisfaction.

The strength of our study is its position as one of the few studies to examine a broader range of macro-level factors in relation to multiple mental health indicators, on top of individual covariates. In summary, we find ED contributes to better depressive status. EI is negatively associated with cognitive functions and life satisfactions. While our findings are not conclusive, they suggest that institutional factors like GWPH and PHRI in China may not be sufficiently supportive of mental health. This highlights the need for further policy considerations.

In the context of population ageing in China, integrating macro-level factors into health policies can significantly enhance depression management, cognitive health, and life satisfaction. For economic development (ED), policies should focus on increasing pensions and providing financial assistance for healthcare and daily living, reducing economic stress—a key factor in late-life depression. Economic growth should also support community-based programme that foster social engagement, which can improve life satisfaction and protect against cognitive decline. Addressing economic inequality (EI) is crucial, as disparities exacerbate mental health challenges among older adults. Policies should aim to ensure equitable access to quality healthcare, particularly mental health and cognitive care. This could involve targeted subsidies for low-income seniors and policies that promote social participation, helping to mitigate feelings of isolation and enhance life satisfaction.

Government healthcare expenditure (GWPH) should prioritise geriatric mental health services, including funding for routine cognitive screenings and depression assessments. Expanding access to psychotherapy, cognitive rehabilitation, and community mental health services will support older adults in maintaining cognitive function and emotional well-being. Investments in public health-related infrastructure (PHRI) should focus on creating age-friendly environments, such as accessible healthcare facilities, day-care centres for seniors, and telehealth systems tailored to their needs. These infrastructures can facilitate preventive care, early detection of mental health issues, and ongoing support, improving both life satisfaction and overall mental health outcomes.

Incorporating these factors into health policies requires a balanced approach. Economic development should focus on not only growth but also equitable access to resources and services. By aligning economic policies with mental health needs and targeting vulnerable populations, it is possible to reduce the risk of depression and improve overall well-being in China’s ageing population.

## Data Availability

Publicly available datasets were analysed in this study. This data can be found at: http://charls.pku.edu.cn/en/Data/Harmonized_CHARLS.htm.

## References

[1] Ding X Billari FC Gietel-Basten S. Health of midlife and older adults in China: the role of regional economic development, inequality, and institutional setting. Int J Public Health. (2017) 62:857–67. doi: 10.1007/s00038-017-0970-9, PMID: 28434029 PMC5641278

[2] Anand S Ravallion M. Human development in poor countries: on the role of private incomes and public services. J Econ Perspect. (1993) 7:133–50. doi: 10.1257/jep.7.1.133

[3] Pickett K Wilkinson R. The spirit level: Why equality is better for everyone. UK: Penguin (2010).

[4] Preston SH. The changing relation between mortality and level of economic development. Population Stud. (1975) 29:231–48. doi: 10.1080/00324728.1975.10410201 11630494

[5] World Health Organization. China country assessment report on ageing and health (2015). Geneva, Switzerland: World Health Organization.

[6] Ng SW Norton EC Popkin BM. Why have physical activity levels declined among Chinese adults? Findings from the 1991–2006 China health and nutrition surveys. Soc Sci Med. (2009) 68:1305–14. doi: 10.1016/j.socscimed.2009.01.035, PMID: 19232811 PMC2731106

[7] Sherif S Sumpio BE. Economic development and diabetes prevalence in MENA countries: Egypt and Saudi Arabia comparison. World J Diabetes. (2015) 6:304. doi: 10.4239/wjd.v6.i2.304 25789111 PMC4360423

[8] Bossert T Chitah MB Bowser D. ‘Decentralization in Zambia: resource allocation and district performance’. Health Policy Plan. (2003) 18:357–69. doi: 10.1093/heapol/czg044 14654512

[9] Gilson L Mills A. Health sector reforms in sub-Saharan Africa: lessons of the last 10 years. Health Policy. (1995) 32:215–43. doi: 10.1016/0168-8510(95)00737-D 10156640

[10] Mills A. ‘Decentralization and accountability in the health sector from an international perspective: what are the choices?’. Public Adm Dev. (1994) 14:281–92. doi: 10.1002/pad.4230140305

[11] Peckham S Exworthy M Greener I Powell M. ‘Decentralizing health services: more local accountability or just more central control?’. Public Money Manag. (2005) 25:221–8. doi: 10.1080/09540962.2005.10600097

[12] Maharani A Tampubolon G. Has decentralisation affected child immunisation status in Indonesia? Glob Health Action. (2014) 7:24913. doi: 10.3402/gha.v7.24913, PMID: 25160515 PMC4164015

[13] Verhaeghe P-P Tampubolon G. Individual social capital, neighbourhood deprivation, and self-rated health in England. Soc Sci Med. (2012) 75:349–57. doi: 10.1016/j.socscimed.2012.02.057, PMID: 22560798

[14] Truesdale BC Jencks C. Income inequality and health: strong theories, weaker evidence. Front Public Health Serv Syst Res. (2016) 5:30–7. doi: 10.13023/FPHSSR.0505.05

[15] Biggs B King L Basu S Stuckler D. Is wealthier always healthier? The impact of national income level, inequality, and poverty on public health in Latin America. Soc Sci Med. (2010) 71:266–73. doi: 10.1016/j.socscimed.2010.04.002 20471147

[16] Rajan K Kennedy J King L. ‘Is wealthier always healthier in poor countries? The health implications of income, inequality, poverty, and literacy in India’. Soc Sci Med. (2013) 88:98–107. doi: 10.1016/j.socscimed.2013.04.004 23702215

[17] Muramatsu N. ‘County-level income inequality and depression among older Americans’. Health Serv Res. (2003) 38:1863–84. doi: 10.1111/j.1475-6773.2003.00206.x 14727801 PMC1360977

[18] Wang Q Granados JAT. ‘Economic growth and mental health in 21st century China’. Soc Sci Med. (2019) 220:387–95. doi: 10.1016/j.socscimed.2018.11.031 30529490

[19] Graham C. Happiness around the world: The paradox of happy peasants and miserable millionaires. Oxford, UK: Oxford University Press (2012).

[20] Piketty T. Capital in the twenty-first century. Cambridge, Massachusetes, USA: Harvard University Press (2014).

[21] Ruhm CJ. ‘Health effects of economic crises’. Health Econ. (2016) 25:6–24. doi: 10.1002/hec.3373 27870301

[22] Frohman EM. ‘A psycho-neuro-endocrine framework for depression: a clinically eclectic approach’. J Mind Behav. (1984) 1:151–69.

[23] Park DC Nisbett RE Hedden T. Culture, cognition, and aging. J Gerontol B. (1999) 54B:P75–84. doi: 10.1093/geronb/54B.2.P75 10097769

[24] Schaie KW Hofer SM. Longitudinal studies in aging research. Academic Press, San Diego, Califonia, USA: Academic Press (2001).

[25] Skirbekk V Stonawski M Bonsang E Staudinger UM. The Flynn effect and population aging. Intelligence. (2013) 41:169–77. doi: 10.1016/j.intell.2013.02.001

[26] Verhaeghen P Salthouse TA. Meta-analyses of age–cognition relations in adulthood: estimates of linear and nonlinear age effects and structural models. Psychol Bull. (1997) 122:231–49. doi: 10.1037/0033-2909.122.3.231 9354147

[27] le Carret N Lafont S Letenneur L Dartigues JF Mayo W Fabrigoule C. The effect of education on cognitive performances and its implication for the constitution of the cognitive reserve. Dev Neuropsychol. (2003) 23:317–37. doi: 10.1207/S15326942DN2303_1, PMID: 12740188

[28] Kim D Griffin BA Kabeto M Escarce J Langa KM Shih RA. ‘Lagged associations of metropolitan statistical area-and state-level income inequality with cognitive function: the health and retirement study’. PLoS One. (2016) 11:e0157327. doi: 10.1371/journal.pone.0157327 27332986 PMC4917220

[29] Elias MF Elias PK Sullivan LM Wolf PA D'Agostino RB. ‘Lower cognitive function in the presence of obesity and hypertension: the Framingham heart study’. Int J Obes. (2003) 27:260–8. doi: 10.1038/sj.ijo.802225 12587008

[30] Diener E Napa Scollon C Lucas RE In: Diener E, editor. The evolving concept of subjective well-being: the multifaceted nature of happiness’, assessing well-being. Heidelberg, Germany: Springer (2009). 67–100.

[31] Kessler E-M Staudinger UM. Intergenerational potential: effects of social interaction between older adults and adolescents. Psychol Aging. (2007) 22:690–704. doi: 10.1037/0882-7974.22.4.690 18179289

[32] Headey B Kelley J Wearing A. ‘Dimensions of mental health: life satisfaction, positive affect, anxiety and depression’. Soc Indic Res. (1993) 29:63–82. doi: 10.1007/BF01136197

[33] Alesina A Di Tella R MacCulloch R. Inequality and happiness: are Europeans and Americans different? J Public Econ. (2004) 88:2009–42. doi: 10.1016/j.jpubeco.2003.07.006

[34] Schwarze J Härpfer M. Are people inequality averse, and do they prefer redistribution by the state?: evidence from german longitudinal data on life satisfaction. J Socio-Econ. (2007) 36:233–49. doi: 10.1016/j.socec.2005.11.047

[35] Verme P. ‘Life satisfaction and income inequality’. Rev Income Wealth. (2011) 57:111–27. doi: 10.1111/j.1475-4991.2010.00420.x

[36] Oishi S Kesebir S Diener E. ‘Income inequality and happiness’. Psychol Sci. (2011) 22:1095–100. doi: 10.1177/0956797611417262 21841151

[37] Oshio T Kobayashi M. ‘Area-level income inequality and individual happiness: evidence from Japan’. J Happiness Stud. (2011) 12:633–49. doi: 10.1007/s10902-010-9220-z

[38] Tomes N. Income distribution, happiness and satisfaction: a direct test of the interdependent preferences model. J Econ Psychol. (1986) 7:425–46. doi: 10.1016/0167-4870(86)90032-2

[39] Clark A. Inequality-aversion and income mobility. A direct test. Delta. Paris, France: Citeseer (2003).

[40] Tomioka J Ohtake F. Happiness and income inequality in Japan. Mimeo: Osaka University (2004).

[41] Hirschman AO Rothschild M. ‘The changing tolerance for income inequality in the course of economic development: with a mathematical appendix’. Q J Econ. (1973) 87:544–66. doi: 10.2307/1882024

[42] Senik C. When information dominates comparison: learning from Russian subjective panel data. J Pub Econ. (2004) 88:2099–123. doi: 10.1016/S0047-2727(03)00066-5

[43] Smyth R Qian X. Inequality and happiness in urban China. Econ Bulletin Citeseer. (2008) 4:1–10.

[44] Knight J Gunatilaka R. ‘The rural–urban divide in China: income but not happiness?’. J Dev Stud. (2010) 46:506–34. doi: 10.1080/00220380903012763

[45] Jiang S Lu M Sato H. Identity, inequality, and happiness: evidence from urban China. World Dev. (2012) 40:1190–200. doi: 10.1016/j.worlddev.2011.11.002

[46] Yang W Hu B. ‘Catastrophic health expenditure and mental health in the older Chinese population: the moderating role of social health insurance’. J Gerontol: Series B. (2022) 77:160–9. doi: 10.1093/geronb/gbab130 PMC8755894 34255044

[47] Minicuci N Naidoo N Corso B Rocco I Chatterji S Kowal P. ‘Data resource profile: cross-national and cross-study sociodemographic and health-related harmonized domains from SAGE plus CHARLS, ELSA, HRS, LASI and SHARE (SAGE+ wave 2)’. Int J Epidemiol. (2019) 48:14–14j. doi: 10.1093/ije/dyy227 30508091 PMC6380306

[48] Zhao Y Hu Y Smith JP Strauss J Yang G. ‘Cohort profile: the China health and retirement longitudinal study (CHARLS)’. Int J Epidemiol. (2014) 43:61–8. doi: 10.1093/ije/dys203 23243115 PMC3937970

[49] Organization, W. H. World mental health report: transforming mental health for all. Geneva: World Health Organization (2022).

[50] Boey KW. ‘Cross-validation of a short form of the CES-D in Chinese elderly’. Int J Geriatric Psychiatry. (1999) 14:608–17. 10.1002/(sici)1099-1166(199908)14:8<608::aid-gps991>3.0.co;2-z 10489651

[51] Chen H Mui AC. Factorial validity of the Center for Epidemiologic Studies Depression Scale short form in older population in {China}. Int Psychogeriatr. (2014) 26:49–57. doi: 10.1017/S1041610213001701, PMID: 24125553

[52] Lam CLK Tse EYY Gandek B Fong DYT. The SF-36 summary scales were valid, reliable, and equivalent in a Chinese population. J Clin Epidemiol. (2005) 58:815–22. doi: 10.1016/j.jclinepi.2004.12.008, PMID: 16018917

[53] Zeng J Jian W. Changes in income-related inequalities of depression prevalence in China: a longitudinal, population study. Soc Psychiatry Psychiatr Epidemiol. (2019) 54:1133–42. doi: 10.1007/s00127-019-01710-0, PMID: 31004180

[54] Crimmins EM. Assessment of cognition using surveys and neuropsychological assessment: the health and retirement study and the aging, demographics, and memory study. J Gerontol B Psychol Sci Soc Sci. (2011) 66:i162–71. doi: 10.1093/geronb/gbr087 21743047 PMC3165454

[55] Gatz M Schneider S Meijer E Darling JE Orriens B Liu Y. ‘Identifying cognitive impairment among older participants in a nationally representative internet panel’. J Gerontol: Series B. (2023) 78:201–9. doi: 10.1093/geronb/gbac172 PMC9938921 36308489

[56] Chen X Fan X Zhao L Duan L Wang Z Han Y. Telephone-based cognitive screening for stroke patients in China. Int Psychogeriatr. (2015) 27:2079–85. doi: 10.1017/S1041610215000551, PMID: 25881853

[57] Meng C Zhang X Zhou J. Telephone interview for cognitive status-modified in screening dementia. Chin Ment Health J. (1992)

[58] Evandrou M Falkingham J Feng Z Vlachantoni A. ‘Individual and province inequalities in health among older people in China: evidence and policy implications’. Health Place. (2014) 30:134–44. doi: 10.1016/j.healthplace.2014.08.009 25262491

[59] Thomas V Wang Y Fan X. Measuring education inequality: Gini coefficients of education. Policy Res. (2001) 2525.

[60] Sundrum RM. Income distribution in less developed countries. London, UK: Routledge (2004).

[61] Azur MJ Stuart EA Frangakis C Leaf PJ. ‘Multiple imputation by chained equations: what is it and how does it work?’. Int J Methods Psychiatr Res. (2011) 20:40–9. doi: 10.1002/mpr.329 21499542 PMC3074241

[62] Cutler DM Katz LF. Rising inequality? Changes in the distribution of income and consumption in the 1980s In: National Bureau of economic research. Cambridge, USA: Mass (1992)

[63] Deaton A Zaidi S. Guidelines for constructing consumption aggregates for welfare analysis. Washington, D.C., USA: World Bank Publications (2002).

[64] Hanandita W Tampubolon G. Does poverty reduce mental health? An instrumental variable analysis. Soc Sci Med. (2014) 113:59–67. doi: 10.1016/j.socscimed.2014.05.005 24836844

[65] Poterba JM. Lifetime incidence and the distributional burden of excise taxes. Mass., USA: National Bureau of Economic Research Cambridge (1989).

[66] Xian W Xu X Li J Sun J Fu H Wu S. Health care inequality under different medical insurance schemes in a socioeconomically underdeveloped region of China: a propensity score matching analysis. BMC Public Health BioMed Central. (2019) 19:1–9. doi: 10.1186/s12889-019-7761-6 PMC6815066 31653250

[67] Xie Y Zhou X. ‘Income inequality in today’s China’. Proc Natl Acad Sci. (2014) 111:6928–33. doi: 10.1073/pnas.1403158111, PMID: 24778237 PMC4024912

[68] Subramanian SV Kawachi I. Income inequality and health: what have we learned so far? Epidemiol Rev. (2004) 26:78–91. doi: 10.1093/epirev/mxh003, PMID: 15234949

[69] Blakely TA Kennedy BP Glass R Kawachi I. What is the lag time between income inequality and health status? Epidemiol Community Health. (2000) 54:318–9. doi: 10.1136/jech.54.4.318, PMID: 10827916 PMC1731662

[70] Stern Y. ‘What is cognitive reserve? Theory and research application of the reserve concept’. J Int Neuropsychol Soc. (2002) 8:448–60. doi: 10.1017/S1355617702813248 11939702

[71] Tucker-Drob EM Johnson KE Jones RN. The cognitive reserve hypothesis: a longitudinal examination of age-associated declines in reasoning and processing speed. Dev Psychol. (2009) 45:431–46. doi: 10.1037/a0014012 19271829 PMC3230274

[72] Easterlin RA. ‘Does economic growth improve the human lot? Some empirical evidence’. Nations Households Econ Growth. (1974) 1:89–125. doi: 10.1016/B978-0-12-205050-3.50008-7

[73] Appleton S Song L. Life satisfaction in urban China: components and determinants. World Dev. (2008) 36:2325–40. doi: 10.1016/j.worlddev.2008.04.009

[74] Easterlin RA Wang F Wang S. Growth and happiness in China, 1990–2015 In: A modern guide to the economics of happiness. Ed. Lars E. Cheltenham, UK: Edward Elgar Publishing (2021)

[75] Wang P Pan J Luo Z. The impact of income inequality on individual happiness: evidence from China. Soc Indic Res. (2015) 121:413–35. doi: 10.1007/s11205-014-0651-5

[76] Wu X Li J. Economic growth, income inequality and subjective well-being: Evidence from China Population Studies Center Research Report (2013). 13:29.

[77] Gao J. Health equity in transition from planned to market economy in China. Health Policy Plan. (2002) 17:20–9. doi: 10.1093/heapol/17.suppl_1.20 12477738

[78] Yip WC-M Hsiao WC. Non-evidence-based policy: how effective is China’s new cooperative medical scheme in reducing medical impoverishment?’. Health Care Policy East Asia: World Scientific Reference: Volume 1: Health Care System Reform Policy Res China. (2020) 1:85–105.

[79] Bank, W. Live long and prosper: Aging in East Asia and Pacific. Washingto, D.C., USA: The World Bank (2015).

[80] Li J Raine JW. The time trend of life satisfaction in China. Soc Indic Res. (2014) 116:409–27. doi: 10.1007/s11205-013-0300-4

[81] Sun J. ‘Equity in access to healthcare among the urban elderly in China: does health insurance matter?’. Int J Health Plann Manag. (2014) 29:e127–44. doi: 10.3390/ijerph18158056 25028751

[82] Ng ST Tey NP Asadullah MN. What matters for life satisfaction among the oldest-old? Evidence from China. PLoS One. (2017) 12:e0171799. doi: 10.1371/journal.pone.0171799, PMID: 28187153 PMC5302476

[83] Liang D Mays VM Hwang W-C. ‘Integrated mental health services in China: challenges and planning for the future’. Health Policy Plan. (2018) 33:107–22. doi: 10.1093/heapol/czx137 29040516 PMC5886187

[84] Meng Q Fang H Liu X Yuan B Xu J. Consolidating the social health insurance schemes in China: towards an equitable and efficient health system. Lancet. (2015) 386:1484–92. doi: 10.1016/S0140-6736(15)00342-6 26466052

[85] Zhang C Lei X Strauss J Zhao Y. Health insurance and health care among the mid-aged and older Chinese: evidence from the national baseline survey of CHARLS. Health Econ. (2017) 26:431–49. doi: 10.1002/hec.3322 26856894 PMC4980285

[86] Carlsson M Hamrin E. Evaluation of the life satisfaction questionnaire (LSQ) using structural equation modelling (SEM). Qual Life Res. (2002) 11:415–26. doi: 10.1023/A:1015670628990 12113389

[87] Wang J Zhang J Lin H Han Y Tu J Nie X. ‘Economic development, weak ties, and depression: evidence from China’. J Affect Disord. (2023) 334:246–57. doi: 10.1016/j.jad.2023.04.097 37146909

[88] Sun J Wang S Zhang JQ Li W. Assessing the cumulative effects of stress: the association between job stress and allostatic load in a large sample of Chinese employees. Work Stress. (2007) 21:333–47. doi: 10.1080/02678370701742748

