# Supplementary material for: Macro-level socioeconomic factors and mental health in midlife and older adults in China: a multilevel analysis
Source: Front Public Health. 2024 Dec 23;12:1435263. doi: 10.3389/fpubh.2024.1435263 (PMC11701142; doi:10.3389/fpubh.2024.1435263)
Supplement: Supplementary file 1 [file Data_Sheet_1.PDF]

frontiers

Macro-Level Socioeconomic factors and Mental Health in Midlife and Older Adults in China: A Multilevel Analysis

Supplementary Material

Supplementary Table S1: Summary of literature: Macro-level factors influencing mental health and well-being

	Authors, date	Location	Demographics of participants	Number of participants	Macro-level effect on mental health and well-being
1	Ding, Billari and Gietel-Basten, 2017	China	Middle-age and older adults (45+)	11,635	-Health is measured by allostatic load, an index indicating stress and physiological dysregulation.<br>-Economic growth is associated with better self-rated health, but also with obesity.<br>-Better health infrastructure tends to be negatively associated with health outcomes, indicating the likely presence of reverse causality.<br>-No supportive evidence found that income inequality leads to worse health outcomes.
2	Wang and Granados, 2019	China	Middle-age and older adults (45+)	over 17,000	The cross-sectional study finds that higher rates of economic growth are associated with poorer depressive status, worse cognitive capacities and life dissatisfaction.
3	Ruhm, 2016	USA	All age	118,326 observations from 3142 counties.	Severe economic recessions offset the predicted rate of suicides
4	Le Carret et al., 2003	France	66 years old + without physical or neurological disorders	1,022	Education specifically increases 2 cognitive components: controlled processes and conceptualization ability. Moreover, mental stimulation occurring after the education years, such as high-complex activity occupations, seems to increase the controlled component.
5	Alesina, Di Tella and MacCulloch, 2004	Europe<br>USA	Adults	123,668	Individuals have a lower tendency to report themselves happy when inequality (measured by Gini coefficient) is high, even after controlling for individual income, a large set of personal characteristics, and year and country (or, in the case of the US, state) dummies.
6	Schwarze and Härpfer, 2007	German	West German population of working age (20-60 years old)	approximately 6,000 families in the western states at baseline (1984)	Inequality reduction by the state does not increase well-being. On the contrary, inequality reduction imposes an excess burden on middle-income earners

7	Verme, 2011	Europe	Mean age = 41.24, SD = 16.33	a total of 267,870 individuals across 4 waves	Income inequality has a negative and significant effect on life satisfaction.
8	Oishi, Kesebir and Diener, 2011	USA	18-89 years old	53043	Americans were on average happier in the years with less national income inequality than in the years with more national income inequality. We further demonstrated that this inverse relation between income inequality and happiness was explained by perceived fairness and general trust.
9	Oshio and Kobayashi, 2011	Japan	26-80 years old	4,393	People live in areas of high-income inequality tend to report themselves as being less happy, even after controlling for various individual and area-level factors.
10	Tomes, 1986	Canada	Mean age = 42.12	3267	self-reported satisfaction is lower the larger the share of income going to the poorest 40% of the community.
11	Clark, 2003	UK	Working age (16-60 years old)	approximately 10,000 individuals in 5,500 British households	Preference for inequality is strongest among individuals whose incomes have fluctuated the most over the past three years and those experiencing the fastest income growth. Income inequality seems to include some aspect of opportunity.
12	Tomioka and Ohtake, 2004	Japan	20-65 years old	1,928	Both the Gini coefficient and perception of a rising inequality have a weak but positive correlation to happiness.
13	Hirschman and Rothschild, 1973	USA	n/a	n/a	People may appreciate inequality (measured by Gini) if it signals social mobility, a phenomenon also called the ‘tunnel effect’
14	Senik, 2004	Russia	Panel data, age not indicated at baseline	4,685	“Tunnel effect” conjecture seems to be confirmed: variables reflecting income distribution do not influence satisfaction through social comparisons. Inequality indices (measured by STARK indices and Gini) do not affect individual welfare.
15	Knight and Gunatilaka, 2010	China	Mean age = 45.41	15973	A higher Gini coefficient in rural counties increases happiness due to the “demonstration effect”
16	Jiang, Lu and Sato, 2012	China	Mean age = 43.31, SD = 11.73	5,630	A positive correlation between city-level Gini coefficients and happiness in China.

Supplementary Table S2. Specification of Instruments

Instruments	Specification
**Marital Status**	Self-reported marital status: 0. Married and live together 1. Separated or married but not live together.
**Education Background**	10-level self-reported school experience. We divided education level into six categories: 5. Illiterate, 4. Sishu (a traditional Chinese school teaching reading and basic knowledge) or can read, 3. Primary school or equivalent, 2. Middle school or equivalent, 1. High school or equivalent, 0. (reference) Graduate degree or higher.
**Residence Type**	Data are automatically obtained based on the location the survey taken place if the participants live in rural or urban.
**Economic Status**	Economic status is calculated based on participants' annual expenditures. The economic status was given to each individual based on the interquartile range one belongs to.
**Insurance**	Participants were asked if they have participated in any kind.<br><br>We categories: 1. Not participate in any kind, 0. Yes.
**Smoking Behaviours**	Participants were asked the quantities of cigarettes they consume daily.<br><br>We categories: 1. current smoke, 0. Never smoke.
**Drinking Behaviours**	Drinking is measured based on frequency instead of quantity in this study.
**ADL**	Participants were asked if they have difficulties with 1. Dressing, 2. Taking bath, 3. Eating, 4. Bedding, 5. Taking toilet and 6. Urinating by themselves. We categories 1. Have difficulties in ADL and, 0. Have no difficulties.
**Num. Comorbidities**	14 comorbidities were included in the study based on medical diagnosis. They are: 1. Hypertension, 2. Dyslipidemia, 3. Diabetes or High Blood Sugar, 4. Cancer or Malignant Tumour, 5. Chronic Lung Diseases, 6. Liver Disease, 7. Heart Problems, 8. Stroke, 9. Kidney Disease, 10. Stomach or Other Digestive Disease, 11. Emotional, Nervous, or Psychiatric Problems, 12. Memory-Related Disease, 13. Arthritis or Rheumatism and 14. Asthma.

Table S3. The coefficients and standard errors of two-level mixed effects models for mental health indicators, H-CHARLS 2018

	Depressive Symptoms	Cognitive Functions	Life Satisfaction
	Model 1	Model 2	Model 3
**Economic Development**	-0.34 (0.06) ***	-0.04 (0.08)	0.00 (0.03)
**Economic Inequality**	-0.15 (3.16)	-8.82 (4.01) *	-0.74 (0.39) †
**GWPH**	-0.07 (0.13)	-0.07 (0.16)	-0.03 (0.04)
**PHRI**	-0.14 (0.18)	-0.09 (0.20)	-0.02 (0.05)
Age	-0.06 (0.01) ***	-0.15 (0.01) ***	0.00 (-0.01) ***
Female (ref=male)	1.39 (0.06) ***	0.33 (0.03) ***	-0.04 (0.02) ***
Rural resident (ref=urban)	1.13 (0.03) ***	-1.07 (0.03) ***	-0.01 (0.00) ***
Single/divorced/separated/widows (ref=married or live together)	1.10 (0.07) ***	-0.68 (0.04) ***	-0.11 (0.02) ***
Economic status (ref=highest)			
2ed quartile (middle-high)	0.11 (0.07) ***	-0.24 (0.06) ***	-0.04 (0.02) ***
3rd quartile (middle-low)	0.28 (0.05) ***	-0.51 (0.06) ***	-0.06 (0.00) ***
4th quartile (lowest)	0.39 (0.07) ***	-1.06 (0.04) ***	-0.08 (0.02) ***
Education background (ref=college degree or higher)			
High School	0.65 (0.15) ***	-1.12 (0.14) ***	-0.04 (0.03)
Middle School	1.23 (0.16) ***	-2.15 (0.12) ***	-0.02 (0.03)
Primary School	1.48 (0.14) ***	-3.47 (0.14) ***	0.00 (0.03)
Sishu/can read	2.46 (0.17) ***	-6.27 (0.14) ***	-0.01 (0.03)
Illiterate	2.19 (0.15) ***	-10.31 (0.14) ***	0.06 (0.03) †
Have no insurance (ref=have insurance of any kind)	0.15 (0.09) †	-1.14 (0.08) ***	-0.08 (0.00) ***
Current smoker (ref=don't smoke)	0.46 (0.06) ***	-0.64 (0.03) ***	-0.05 (0.02) ***
Drink alcohol (ref=don't drink)	-0.38 (0.04) ***	0.45 (0.05) ***	0.02 (0.02) †
Num. comorbidities (ref=none)			
1	0.79 (0.07) ***	0.38 (0.06) ***	-0.06 (0.00) ***
2	1.53 (0.05) ***	0.33 (0.06) ***	-0.10 (0.02) ***
3 or more	3.13 (0.05) ***	0.56 (0.06) ***	-0.23 (0.00) ***
Have difficulties in ADL (ref=have no ADL)	3.94 (0.04) ***	-0.73 (0.06) ***	-0.23 (0.00) ***
Depression	n/a	-1.39 (0.05) ***	n/a
Constance	5.65 (2.02) **	32.38 (2.50) ***	3.39 (0.44) **
Province-level (level 2) Variance	0.73 (0.09)	0.94 (0.12)	0.17 (0.02)

Note: **Multiple imputation of ten cycles** has been applied to the model
Significant level: †10%, *5%, **1%, ***0.1%

Supplementary Table S4.
Actual values of macro-level factors by Province, 2018

	Province	ED (pc1)	GWPH (pc2)	PHRI (pc3)	EI (Gini) [a]	Gini [b]	Gini[b] – Gini[a]
1	Shanghai	6.014926	-1.697323	-0.6131014	0.35519	0.53	0.17
2	Yunnan	-1.346012	0.9102087	-0.8833987	0.46953	0.67	0.20
3	Inner Mongolia	0.5146369	-2.317786	-0.3976757	0.38658	0.59	0.20
4	Beijing	5.394528	-1.494507	-0.1874288	0.45863	0.46	0.00
5	Jilin	-0.2459067	-1.537592	-0.8825236	0.50861	0.56	0.05
6	Sichuan	-1.591138	-0.2657177	1.626305	0.35821	0.70	0.34
7	Tianjin	5.173717	-1.105324	-2.424778	0.47151	0.60	0.13
8	Anhui	-0.0495141	1.610997	-1.426409	0.3598	0.68	0.32
9	Shandong	0.5835496	0.330709	0.8260389	0.41715	0.61	0.19
10	Shanxi	-0.99758	-0.9309157	-0.8172676	0.4215	0.70	0.28
11	Guangdong	2.896738	2.731452	0.6955788	0.45105	0.63	0.18
12	Guangxi	-1.305128	1.522894	-1.324496	0.40085	0.60	0.20
13	Xinjiang	-0.5865253	-2.781466	-0.3497083	0.3863	0.55	0.16
14	Jiangsu	3.350609	-0.230374	0.6191511	0.37359	0.58	0.21
15	Jiangxi	-0.9293962	1.223667	-0.8357295	0.49351	0.68	0.19
16	Hebei	-1.080174	0.048273	0.0696217	0.47777	0.61	0.13
17	Henan	-1.180707	1.27138	0.772471	0.38973	0.59	0.20
18	Zhejiang	2.552169	-0.632388	-0.1551695	0.46159	0.66	0.20
19	Hubei	0.4502738	-0.7801122	0.1217643	0.37672	0.62	0.24
20	Hunan	-0.790091	-0.8368707	0.6379787	0.46887	0.68	0.21
21	Gansu	-2.218223	-1.037004	-0.8374925	0.35508	0.62	0.26
22	Fujian	1.56372	0.4766328	-1.258082	0.46386	0.71	0.25
23	Guizhou	-2.044526	-0.1124153	-0.1088381	0.46604	0.66	0.19
24	Liaoning	0.5957133	-2.709203	0.1986341	0.45687	0.58	0.12
25	Chongqing	0.420442	-1.474326	0.0350682	0.45277	0.62	0.17
26	Shaanxi	-0.5050167	-0.9746544	0.0802409	0.3712	0.58	0.21
27	Qinghai	-1.571816	-1.61338	-0.8082679	0.47202	0.57	0.10
28	Heilongjiang	0.282678	-1.882312	-0.9928615	0.46236	0.60	0.14

Note: [a] gini coefficients are calculated based on stratified regional average individual income from Chinese Statistical Yearbook,
[b] gini coefficients are calculated based on household expenditures from CHARLS 2018.
Gini[b] – Gini[a]: the gini coefficients estimated by household expenditure from CHARLS generally overestimate 0.2 points higher than the other (the mean is 0.19, Sd=0.07). It reflects the household expenditures inequality are bigger compared to individual income inequity at the provincial level. Pearson correlation shows Gini [a] and Gini [b] are positively correlated (r=0.078, p<0.000).

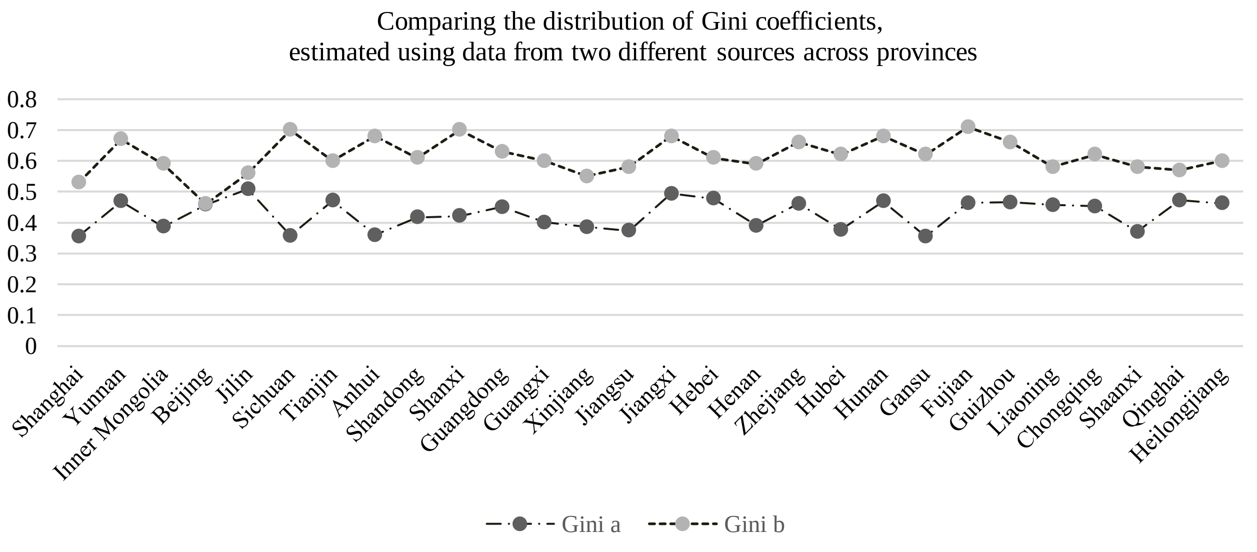

Supplementary Table S5. The coefficients and standard errors of two-level mixed effects models for mental health indicators, H-CHARLS 2018

	Depressive Symptoms	Cognitive Functions	Life Satisfaction
	Model 1	Model 2	Model 3
**Economic Development**	-0.34 (0.07) ***	-0.04 (0.09)	0 (0.02)
**Economic Inequality**	0.33 (3.11)	-9.2 (4.06) *	-0.69 (0.68)
**GWPH**	-0.13 (0.11)	-0.04 (0.15)	-0.04 (0.02) †
**PHRI**	-0.19 (0.15)	0.02 (0.19)	-0.01 (0.03)
**Age**	-0.07 (0) ***	-0.14 (0) ***	0.01 (0) ***
**Female** (ref=male)	1.38 (0.05) ***	0.32 (0.04) ***	-0.03 (0.01) ***
**Rural resident** (ref=urban)	1.14 (0.04) ***	-1.08 (0.04) ***	-0.02 (0.01) **
**Single/divorced/separated/widows** (ref=married or live together)	1.11 (0.06) ***	-0.67 (0.05) ***	-0.1 (0.01) ***
**Economic status** (ref=highest)			
2^ed^ quartile (middle-high)	0.12 (0.06) *	-0.25 (0.05) ***	-0.05 (0.01) ***
3rd quartile (middle-low)	0.29 (0.06) ***	-0.5 (0.05) ***	-0.07 (0.01) ***
4th quartile (lowest)	0.4 (0.06) ***	-1.07 (0.05) ***	-0.07 (0.01) ***
**Education background** (ref=college degree or higher)			
High School	0.64 (0.16) ***	-1.13 (0.13) ***	-0.05 (0.02) *
Middle School	1.24 (0.15) ***	-2.14 (0.13) ***	-0.03 (0.02)
Primary School	1.49 (0.15) ***	-3.46 (0.13) ***	-0.01 (0.02)
Sishu/can read	2.45 (0.16) ***	-6.26 (0.13) ***	0 (0.02)
Illiterate	2.2 (0.16) ***	-10.3 (0.13) ***	0.05 (0.02) *
**Have no insurance** (ref=have insurance of any kind)	0.13 (0.1)	-1.15 (0.09) ***	-0.09 (0.01) ***
**Current smoker** (ref=don't smoke)	0.45 (0.05) ***	-0.63 (0.04) ***	-0.04 (0.01) ***
**Drink alcohol** (ref=don't drink)	-0.39 (0.05) ***	0.44 (0.04) ***	0.01 (0.01) †
**Num. comorbidities** (ref=none)			
1	0.78 (0.06) ***	0.39 (0.05) ***	-0.07 (0.01) ***
2	1.54 (0.06) ***	0.33 (0.05) ***	-0.11 (0.01) ***
3 or more	3.12 (0.06) ***	0.55 (0.05) ***	-0.24 (0.01) ***
**Have difficulties in ADL** (ref=have no ADL)	3.93 (0.05) ***	-0.72 (0.05) ***	-0.25 (0.01) ***
**Depression**	n/a	-1.4 (0.04) ***	n/a
**Constance**	5.31 (1.98) **	32.65 (2.57) ***	3.34 (0.43) ***
Province-level (level 2) Variance	0.70 (0.10)	0.94 (0.13)	0.16 (0.02)
AIC/BIC	588232/588478	559606/559861	217482/217728

Note: EI (gini coefficients) is calculated based on ***household expenditure*** *from CHARLS.*
*Sig level: †10%, *5%, **1%, ***0.1%*
